# Supplementary material for: Automated computed tomography based parasitoid detection in mason bee rearings
Source: PLoS One. 2022 Oct 13;17(10):e0275891. doi: 10.1371/journal.pone.0275891 (PMC9560145; doi:10.1371/journal.pone.0275891)
Supplement: Attachment — Submitted filename: 20220922_response_to_reviewers_final.docx [file pone.0275891.s002.docx]

| Emily Chenette, PhD  Editor-in-Chief  PLOS ONE |  |
| --- | --- |
|  | **Dr. sc. med. vet. Henning Richter**  DVM-PhD, Dipl. SVLAS  Telefon +41 44 635 86 75  henning.richter@uzh.ch |
| Zürich, Sept 22 2022 | |
|  | |

Response to the reviewers

Dear Emily Chenette, Editor-in-Chief PLOS ONE,

Dear reviewers and editors of PLOS ONE,

We very much appreciate the feedback and time taken by the editor and reviewer. We have prepared careful revisions with regard to the reviewers’ concerns and have highlighted the respective changes in the manuscript. Please find a response below to the point raised by reviewer #2.

**Reviewer #2:**

I believe the authors have adequately addressed my comments. However, one more thing need to be addressed. Insect do not hibernate. They are in diapause; this needs to be corrected throughout the manuscript.

We would like to thank the reviewer for the additional time taken to handle our manuscript – we have made the corrections on page 3 and 4 of our manuscript.

We are very much looking forward to your reply,

Best regards on behalf of all authors,

Bart Thomson and Henning Richter
